# Spatiotemporal changes in the carbon balance of terrestrial ecosystems and analysis of driving factors: A case study of Hainan Province

**DOI:** 10.1016/j.isci.2025.113866

**Published:** 2025-10-27

**Authors:** Qiaoru Ye, Xiaomin Xiao, Qiwen Yu, Chenhao Zhao, Yichen Yan, Yuxin Qi, Ruiming Xiao, Xuechao Wang, Xiaobin Dong

**Affiliations:** 1 State Key Laboratory of Earth Surface Processes and Resource Ecology, Beijing Normal University, Beijing 100875, China; 2 Faculty of Geographical Science, Beijing Normal University, Beijing 100875, China

**Keywords:** Earth sciences, Environmental science, Global Carbon Cycle

## Abstract

Hainan Province is a key region for investigating carbon balance dynamics in tropical ecosystems. This study used the integrated valuation of ecosystem services and tradeoffs (InVEST) model and an improved net ecosystem productivity (NEP) model to simulate the spatial distribution of carbon balance in Hainan Province’s terrestrial ecosystems and identify key driving factors. Results show that carbon balance values are higher in the central region and lower in the periphery, increasing from coastal to inland areas. The central region, with its elevated terrain and dense forest cover, shows greater capacity for carbon absorption and conversion, leading to higher carbon balance. In contrast, coastal areas exhibit lower carbon stocks, mainly due to human disturbances. Land-use changes driven by humans are the primary factor shaping the spatial distribution of carbon balance. Thus, green planning and development should prioritize high carbon-emission areas to restore and enhance ecosystem carbon sink functions.

## Highlights

• Hainan acts as a strong carbon sink, with a sequestration rate of 22.52 Tg C yr^−1^
• Carbon balance shows spatial heterogeneity, increasing from coastal to inland areas
• Human activities drive 71.33% of carbon balance changes, exceeding climate impacts
• Land use is the dominant factor shaping the spatial distribution of carbon balance

## Graphical abstract

**Figure:**
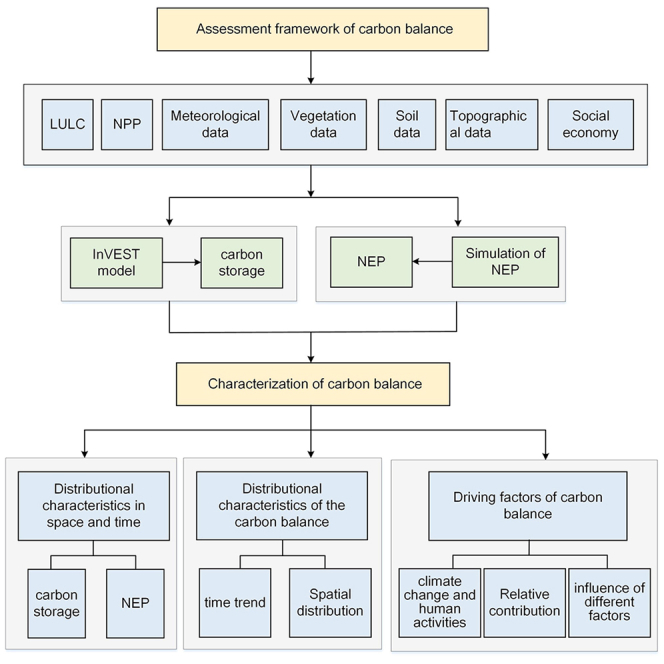

## Introduction

The rapid growth of the global economy and industrialization has significantly altered the natural environment and climate system. According to the IPCC Sixth Assessment Report (AR6), the global surface temperature increased by approximately 1.1°C from 1901 to 2020. Notably, since the late 20th century, the pace of warming has accelerated considerably, posing progressively greater challenges to the global climate system and the ecological environment.^1^ To mitigate the adverse impacts of climate change, countries have broadly agreed on reducing greenhouse gas emissions and enhancing ecosystem carbon storage, both of which are regarded as pivotal strategies to combat climate change.^2^^,^^3^^,^^4^ It has been shown that China’s CO_2_ emissions have increased from 11.35 × 10^8^ t in 1980 to 105.23 × 10^8^ t in 2021, an increase of 93.88 × 10^8^ t in 41 years, and the contribution of industrial CO_2_ emissions to the total CO_2_ emissions is as high as 79.83%.^5^^,^^6^ Under the background of the “double carbon” target, it is urgent to analyze the current situation and change pattern of the carbon balance of China’s ecosystems, and explore the influence mechanisms of various geographic factors and human activities on the carbon balance, so as to provide corresponding suggestions for the low-carbon development of the region. Continuously decreasing carbon emissions and increasing ecosystem carbon sinks to realize sustainable development have become an urgent matter that requires immediate attention.^7^

Carbon is a fundamental element in life processes and has an essential function in ecosystems. Carbon sequestration refers to the process by which carbon is captured and stored for extended durations in various ecosystems. This process occurs primarily through mechanisms such as photosynthesis in plants, the accumulation of soil organic matter, and biogeochemical processes in ecosystems, such as forests, grasslands, and wetlands.^8^ The carbon cycle refers to the process by which carbon flows among different carbon pools. This cycle encompasses multiple stages, including the absorption, storage, transformation, and release of carbon, thereby reflecting the cyclic flow of carbon within ecosystems.^9^ The fundamental concept of carbon balance originally referred to the equilibrium between carbon emissions and absorption, with respect to both quantity and quality. However, as research fields and scopes have expanded, the interpretation of the carbon balance has evolved.^10^ In general, studies of the carbon balance focus on changes in carbon storage and fluxes within ecosystems (carbon storage and net ecosystem productivity (NEP)), aiming to reveal the role of different ecosystems in carbon storage and release.^11^ Understanding the carbon cycle and developing carbon reduction strategies rely on accurate assessments of carbon storage.^12^ Thus, a precise evaluation of regional carbon storage and NEP is essential. Land contains four primary carbon reservoirs: aboveground biomass, belowground biomass, soil organic carbon, and dead organic matter. These reservoirs play key roles in analyzing the spatial distribution of carbon storage.^13^ NEP represents the net CO_2_ absorbed through photosynthesis within an ecosystem and serves as an indicator of the terrestrial carbon source or sink strength.^14^ A positive NEP denotes a carbon sink, whereas a negative NEP indicates a carbon source.

Scholars have conducted extensive and detailed studies on the carbon balance of terrestrial ecosystems, which include studies of the carbon balance and factors influencing it in individual ecosystems, such as forests, wetlands, and grasslands. Li et al. investigated the response of carbon exchange processes in alpine grassland ecosystems to warming and reported that warming has a negative impact on carbon exchange.^15^ Most of the studies have focused on carbon storage, NEP calculations, identification of carbon sources and sinks, and carbon emissions associated with land use. Cheng et al. explored the role of land use changes in driving land carbon cycling and reported that land use changes significantly increased China’s terrestrial carbon sink.^16^ In terms of research methods, the integrated valuation of ecosystem services and tradeoffs (InVEST) model, trajectory analysis, patch-generating land use simulation (PLUS) model, spatial clustering and other methods have been used to measure the carbon balance and its impact. Gao et al. explored carbon balance patterns through ecological support coefficients, social network analysis, and spatial clustering and proposed specific zoning strategies for determining the carbon balance.^2^ Moreover, Gong et al. assessed how land use and land cover changes influence carbon storage in the Nandu River Basin via trajectory analysis and the InVEST model.^17^ In the study of carbon dynamics and related influencing factors in Hainan Province, Zhang et al. applied the InVEST model to investigate the carbon storage capacity of the three major basins in Hainan Island and found that the overall trend of carbon storage is declining, with soil type, slope, and land use intensity identified as the primary driving factors.^18^ Lai et al. analyzed the historical and future changes of carbon storage and the underlying driving forces by combining the InVEST model, the future land use simulation (FLUS) model, and machine learning algorithms, and found that natural factors such as elevation and NDVI are the dominant driving factors, providing a novel framework for carbon management in tropical islands.^19^ All these studies provide a robust theoretical foundation for carbon emission reduction and regional carbon storage management in Hainan Province. However, the potential drivers of the carbon balance remain insufficiently explored.

Investigations of the carbon balance of terrestrial ecosystems are essential for comprehending the dynamics of the global carbon cycle.^20^ The processes of carbon fixation, storage, and release not only affect atmospheric CO_2_ concentrations but also play a critical role in regulating climate stability. Research indicates that terrestrial ecosystems convert CO_2_ into organic carbon via photosynthesis and sequester it in both vegetation and soil, thereby directly impacting global carbon storage.^21^ Moreover, research on the carbon balance can elucidate the mechanisms of carbon cycling within ecosystems, facilitating a more precise evaluation of variations in carbon fluxes and storage across diverse ecosystems. This research further enhances our understanding of how ecosystems respond to climate change and land use alterations.^11^ Additionally, in-depth studies on the carbon balance can reveal the multifaceted functions of terrestrial ecosystems, especially the interplay between carbon storage and ecological processes, such as water and nutrient cycles.^22^ Existing research not only offers crucial insights into ecosystem health assessments but also serves as a scientific foundation for formulating ecological restoration and conservation strategies.^2^^,^^8^^,^^23^ With the intensification of global warming, the role of climate change in shaping the carbon balance of terrestrial ecosystems has become a central research theme.^24^^,^^25^ Simultaneously, policy adjustments,^26^^,^^27^ urban expansion,^4^^,^^10^ and other human activities significantly influence carbon balance dynamics. Thus, analyzing the effects of climate change and anthropogenic factors on vegetation dynamics is essential for strengthening regional climate resilience and guiding national ecological restoration initiatives.

**Figure 1:**
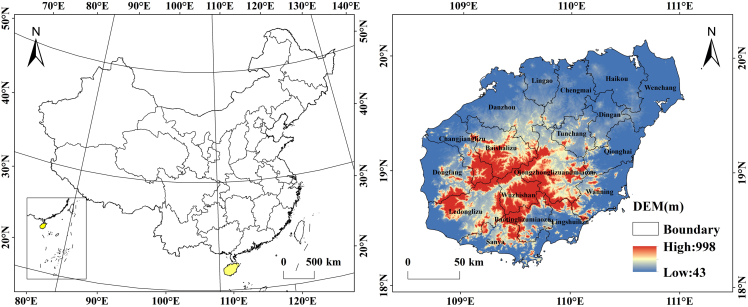
Location and elevation of Hainan Island

Hainan Province, which is designated China’s ecological civilization pilot zone, holds substantial strategic significance in carbon balance research owing to its distinctive geographical and ecological characteristics (Figure 1). Hainan Province not only serves as a model for ecological protection and green development in China but also plays a crucial role in regional climate regulation and carbon storage. The province’s diverse ecosystems, including tropical forests, wetlands, and grasslands, provide it with significant carbon sequestration potential.^28^ However, previous research on Hainan Province has focused primarily on carbon storage,^29^ while studies specifically addressing the carbon balance in Hainan Province, particularly the impacts of climate change and human activities on this balance, remain limited. Investigating Hainan Province’s carbon balance can provide essential data for understanding the processes of carbon storage and release in tropical and subtropical ecosystems. This, in turn, offers robust scientific support for addressing global climate change and guiding the development of regional carbon management policies. Moreover, this research provides valuable insights for formulating carbon management and climate adaptation strategies in other regions.^30^

In this study, the InVEST model and an improved NEP model are used to evaluate the carbon balance of Hainan’s terrestrial ecosystems from 2000 to 2022. By applying correlation analysis, residual analysis, and GeoDetector, the contributions of climate change and human activities to the carbon balance variations are quantitatively examined. Data sources and carbon density parameters used in this study are detailed in Tables 1 and 2, respectively. The overarching goal is to offer scientific support for formulating ecological conservation strategies in Hainan while promoting the high-quality development of national ecological civilization.

**Table 1:** Description of data used in this study

Data	Data attribute	Resolution	Time range	Data source
Land Use/Land Cover (LULC)	CLCD	30 m × 30 m	2000–2022	https://zenodo.org/records/12779975
NPP	Net primary production	500 m × 500 m	2000–2022	https://lpdaac.usgs.gov/
Meteorological factors	Temperature	1 km × 1 km	2000–2022	https://www.geodata.cn/main/face_science_detail?guid=164304785536614&typeName=face_science
	Precipitation	1 km × 1 km	2000–2022	https://www.geodata.cn/main/face_science_detail?guid=192891852410344&typeName=face_science
Vegetation index	NDVI	30 m × 30 m	2000, 2005, 2010, 2015, 2022	https://www.nesdc.org.cn/sdo/detail?id=60f68d757e28174f0e7d8d49
Vegetation factor	Vegetation type	1 km × 1 km	2001	https://www.resdc.cn/data.aspx?DATAID=122
Soil factor	Soil type	1 km × 1 km	1995	https://www.resdc.cn/data.aspx?DATAID=145
	Soil erosion	1 km × 1 km	1995	https://www.resdc.cn/data.aspx?DATAID=259
	Soil organic carbon	1 km × 1 km	1995	https://www.fao.org/soils-portal/data-hub/soil-maps-and-databases/harmonized-world-soil-database-v20/en/
Topographical factors	Elevation	30 m × 30 m	–	https://www.gscloud.cn/
	Slope	30 m × 30 m	–	
	Aspect	30 m × 30 m	–	
Social economy	Population	1 km × 1 km	2000, 2005, 2010, 2015, 2022	https://landscan.ornl.gov/
	GDP	1 km × 1 km	2000, 2005, 2010, 2015, 2020	https://www.resdc.cn/DOI/DOI.aspx?DOIID=33

**Table 2:** Carbon density of each LULC type in Hainan Province

LULC Types	Carbon Density (Mg/hm^2^)			
	C_a_	C_b_	C_s_	C_d_
Cropland	5.72	1.18	96.61	2.10
Forest	19.76	5.3	125.43	2.80
Shrub	4.39	3.67	101.33	0.70
Grassland	4.20	5.84	87.76	1.30
Water	0	0	0	0
Barren	0	0	0.86	0
Impervious	2.60	0.75	34.40	0

C_a_ is the aboveground biomass carbon density; C_b_ is the belowground biomass carbon density; C_s_ is the soil organic carbon density; and C_d_ is the dead organic matter carbon density.

## Results

### Characteristics of changes in carbon storage and the NEP

#### Carbon storage

**Figure 2:**
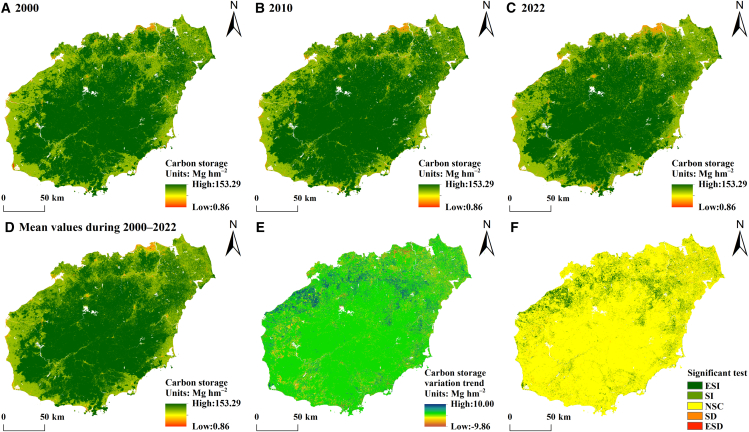
Patterns of the spatial distribution of average carbon storage and trends (A–D) Patterns of the spatial distribution of carbon storage for 2000, 2010, 2022, and the average from 2000 to 2022. (E and F) Patterns of the spatial distribution of carbon storage trends. The ESI, SI, NSC, SD, and ESD represent extremely significant increase, significant increase, no significant change, significant decrease, and extremely significant decrease, respectively.

The carbon storage in Hainan Province from 2000 to 2022 demonstrates significant spatial heterogeneity (Figures 2A–2D). Regions with high carbon storage density are concentrated mainly in the island’s central area, whereas low-density carbon storage zones exhibit a concentric pattern along the coast. The mean carbon storage is 134.99 Mg hm^−2^, with values ranging from 0.86 to 153.29 Mg hm^−2^. On the basis of pixel-scale calculations of the value of the slope trend θ and an F significance test, we analyzed the spatial changes in carbon storage in Hainan Province (Figures 2E and 2F). The θ values for the carbon storage trends in Hainan Province range from −9.86 to 10.00 Mg hm^−2^, with a mean of −0.04 Mg hm^−2^. The area subjected to trend analysis using the F test is 3439.75 km^2^, which constitutes 10.15% of Hainan Island’s total area (Table 3). The expansion of carbon storage surpasses its reduction, with the increase primarily found in northern and northeastern cities in Hainan Province, while the decrease is dispersed across coastal urban areas. Because of the minimal changes in the main land use types, such as forests and cropland, in Hainan Province, the area with no significant changes in carbon storage accounts for as much as 89.85% of the total area. This result indicates that the carbon storage of various ecosystem types in Hainan Province have changed relatively little during the 23-year period, and the terrestrial carbon storage is generally in a relatively stable state. The increase in carbon storage is also an indication that the land use structure is more rational and the ecosystem is in a more benign development.

**Table 3:** Classification of the changes in the average carbon storage and their proportions

Influence level	Abbreviation	Proportion (%)	Area (km^2^)
Extremely significant increase	ESI	7.23	2449.82
Significant increase	SI	1.38	467.78
No significant change	NSC	89.85	30441.53
Significant decrease	SD	1.54	522.15
Extremely significant decrease	ESD	0.00	0.00

#### Net ecosystem productivity

**Figure 3:**
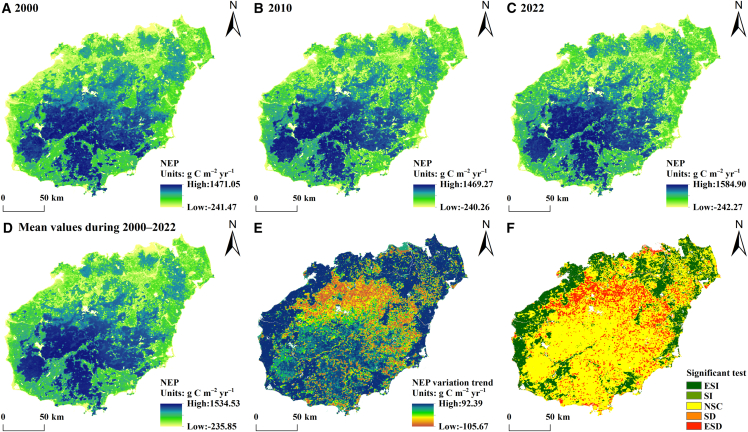
Patterns of the spatial distribution of the NEP and its trends (A–D) Patterns of the spatial distribution of the NEP for 2000, 2010, 2022, and the average from 2000 to 2022. (E and F) Patterns of the spatial distribution of the NEP trend. The ESI, SI, NSC, SD, and ESD represent extremely significant increase, significant increase, no significant change, significant decrease, and extremely significant decrease, respectively.

The annual average NEP distribution in Hainan Province from 2000 to 2022 shows notable spatial variability (Figures 3A–3D). The NEP values are highest in the central regions and decrease toward the periphery, with an increase from coastal to inland areas. This is closely related to the distribution of land use types in Hainan Province, which is dominated by forested land in the central part of the province, with a decrease in forested land in the coastal areas, and the NEP acts mainly on forested land systems, and thus the NEP decreases from the central part of the province to the periphery. The average NEP over this period is 676.68 g C m^−2^ yr^−1^, with values ranging from −244.743 to 1670.99 g C m^−2^ yr^−1^. The average annual carbon sequestration capacity is estimated at 22.52 Tg C yr^−1^. This value suggests that terrestrial ecosystems in Hainan Province are carbon sinks overall, with carbon source areas predominantly located in the developed urban regions along the coast. By conducting pixel-scale calculations of the value of the slope trend θ and performing an F significance test, we analyzed the spatial changes in the annual average NEP in Hainan Province (Figures 3E and 3F). The θ values for the trend analysis of Hainan’s carbon balance of terrestrial ecosystems range from −105.67 to 92.39 g C m^−2^ yr^−1^, with a mean of 0.22 g C m^−2^ yr^−1^. The area subjected to trend analysis using the F test is 14,504.23 km^2^, which constitutes 42.81% of Hainan Island’s total area (Table 4). Regions exhibiting significant increases are located predominantly in coastal cities, such as those in Chengmai County and Haikou City and Wenchang City, whereas areas showing significant decreases are primarily found in the northeastern inland region of Hainan Province. The coastal region, which is in an area of concentrated economic development, has invested more in urban green space and thus has significantly higher NEP levels, while the opposite is true for the northeastern inland region.

**Table 4:** Classification of NEP trend changes and their proportions

Influence level	Abbreviation	Proportion (%)	Area (km^2^)
Extremely significant increase	ESI	21.43	7259.79
Significant increase	SI	5.84	1978.41
No significant change	NSC	57.19	19377.06
Significant decrease	SD	4.94	1672.85
Extremely significant decrease	ESD	10.61	3593.18

### Temporal and spatial variations in carbon balance characteristics

#### Trends in temporal variation

**Figure 4:**
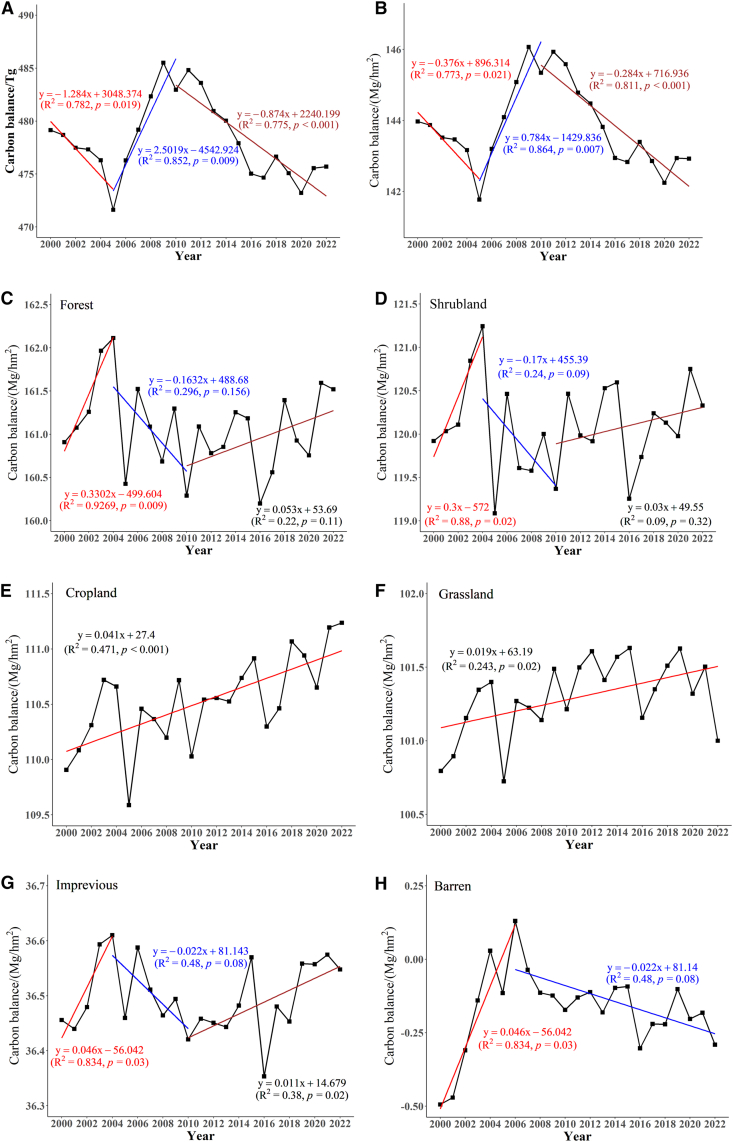
Interannual variation in carbon balance (A) Interannual variation in the total carbon balance in terrestrial ecosystems of Hainan Province (Tg). (B) Interannual variation in the average carbon balance in terrestrial ecosystems (Mg/hm^2^). (C–H) Interannual variation in the carbon balance for forest, shrubland, cropland, grassland, impervious surface, and barren land (Mg/hm^2^) in Hainan Province from 2000 to 2022.

The interannual variation in the carbon balance in the terrestrial ecosystems in Hainan Province from 2000 to 2022 is illustrated in Figures 4A and 4B. The trends exhibit a fluctuating decline from 2000 to 2005 and a significant increase from 2005 to 2010, followed by another period of fluctuating decline from 2010 to 2022. The rates of change in the average carbon balance of terrestrial ecosystems are −0.38 Mg hm^−2^ yr^−1^, 0.78 Mg hm^−2^ yr^−1^, and –0.28 Mg hm^−2^ yr^−1^. The rates of change in the total values are −1.28 Tg yr^−1^, 2.50 Tg yr^−1^, and –0.87 Tg yr^−1^, respectively. The peak carbon balance values and totals occur in 2009, reaching 146.07 Mg hm^−2^ and 485.51 Tg, respectively. Conversely, the lowest values are recorded in 2005, at 141.77 Mg hm^−2^ and 471.62 Tg. The corresponding ranges of fluctuation are 4.30 Mg hm^−2^ and 13.89 Tg. The average carbon balance and interannual variations in various terrestrial ecosystems in Hainan Province from 2000 to 2022 are shown in Figures 4C–4H. Among these ecosystems, forests present the highest average carbon balance, exceeding 160.19 Mg hm^−2^. Shrublands, croplands, and grasslands have average carbon balance values ranging between 100.72 and 121.24 Mg hm^−2^. In contrast, impervious surfaces and bare land have the lowest carbon balance values, ranging from −0.49 to 36.61 Mg hm^−2^. Forests have the highest carbon balance because they have the greatest capacity to absorb and transform carbon; as the amount of green plants decreases, the capacity to absorb and transform carbon decreases, and bare land has the least amount of green plants and biomass, which correspondingly has a particularly low carbon balance. The carbon balance of cropland not only accounts for carbon storage within soil organic pools, but also incorporates the carbon cycling and transformation associated with crop growth by considering the annual cumulative contribution of cropland biomass.^31^ This combined effect results in elevated values compared to studies focusing solely on soil carbon pools, particularly in Hainan’s tropical climate, which enhances biomass accumulation. However, due to rapid biomass turnover through harvesting, the annual contribution of cropland biomass does not represent long-term carbon sequestration.^20^

#### Spatial distribution pattern

**Figure 5:**
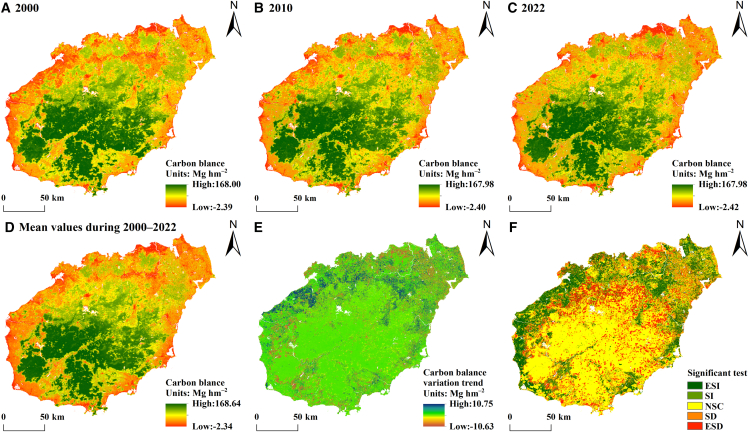
Patterns of the spatial distribution of the average carbon balance and its trend (A–D) Patterns of the spatial distribution of the carbon balance for 2000, 2010, 2022, and the average from 2000 to 2022. (E and F) Patterns of the spatial distribution of the carbon balance trend. The ESI, SI, NSC, SD, and ESD represent extremely significant increase, significant increase, no significant change, significant decrease, and extremely significant decrease, respectively.

The carbon balance in the terrestrial ecosystems in Hainan Province from 2000 to 2022 exhibited significant spatial heterogeneity (Figures 5A–5D). Specifically, the central region displays highly concentrated carbon balance values, whereas lower values are observed around the periphery. There is a clear gradient of increasing carbon balance values from coastal areas toward inland regions. The average carbon balance over this period is 143.84 Mg hm^−2^, with values ranging from −2.34 to 168.64 Mg hm^−2^. The area exhibiting a carbon balance greater than the long-term average constitutes 63.18%, and this area is primarily located in regions such as Baisha Li Autonomous County, Qiongzhong Li and Miao Autonomous County, Wuzhishan City, southeastern Changjiang Li Autonomous County, and eastern Dongfang City in central Hainan, encompassing the Hainan Tropical Rainforest National Park. By conducting pixel-scale calculations of the value of the slope trend θ and performing an F significance test, the trend of spatial changes in the carbon balance in Hainan’s terrestrial ecosystems was analyzed (Figures 5E and 5F). The θ values for the trend analysis of the carbon balance of terrestrial ecosystems in Hainan Province range from −10.63 to 10.75 Mg hm^−2^, with a mean of −0.042 Mg hm^−2^. The area subjected to trend testing by the F test is 15,234.28 km^2^, accounting for 44.96% of Hainan Province’s total area (Table 5). Significant increases are concentrated primarily in coastal agricultural areas of northern Hainan Province, including Dongfang City, Changjiang Li Autonomous County, Danzhou City, and Lingao County. Significant decreases are found mainly in regions at the boundary between inland forests and croplands in Hainan Province. Organic carbon pools of cropland are the most active carbon pools in terrestrial ecosystems, and under the implementation of specific management measures, soil organic carbon pools of cropland increase significantly, resulting in higher carbon balance values in agricultural areas, and a corresponding decrease in the border between cropland and forested areas. Overall, the carbon balance values in Hainan have increased in coastal agricultural zones, decreased in regions at the boundary between inland forests and croplands, and do not significantly change in central forested areas.

**Table 5:** Classification of the changes in the average carbon balance trend and their proportions

Influence level	Abbreviation	Proportion (%)	Area (km^2^)
Extremely significant increase	ESI	18.91	6406.04
Significant increase	SI	4.56	1545.57
No significant change	NSC	55.04	18647.01
Significant decrease	SD	5.28	1787.97
Extremely significant decrease	ESD	16.22	5494.70

### Analysis of driving factors

#### Impact of climate change and human activities on the temporal and spatial evolution of the carbon balance

**Figure 6:**
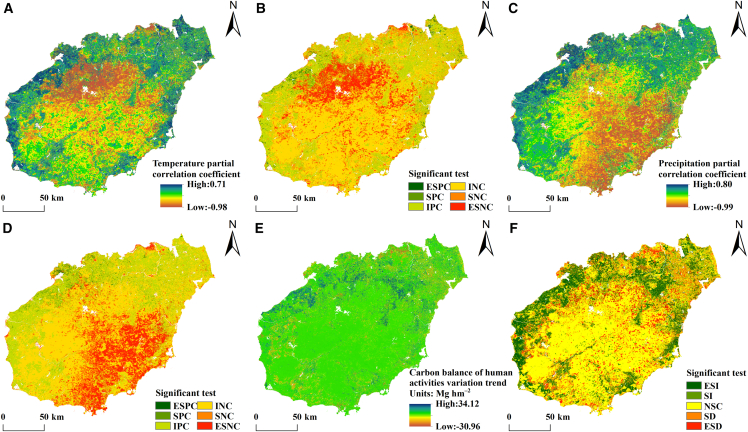
Impacts of climate change and human activities on the spatial and temporal evolution of the carbon balance (A–D) Partial correlation coefficients between the carbon balance and temperature, precipitation, and their corresponding significance tests. (E and F) Impacts of human activities on the carbon balance. ESPC, SPC, IPC, INC, SNC, and ESNC represent extremely significant positive correlation, significant positive correlation, insignificant positive correlation, insignificant negative correlation, significant negative correlation, and extremely significant negative correlation, respectively. The ESI, SI, NSC, SD, and ESD represent extremely significant increase, significant increase, no significant change, significant decrease, and extremely significant decrease, respectively.

The partial correlation coefficients between the carbon balance in Hainan’s terrestrial ecosystems and precipitation and temperature from 2000 to 2022 were calculated at the pixel level and tested using a *t* test (Figures 6A–6D). The results indicate that the partial correlation coefficient between the carbon balance in the terrestrial ecosystems in Hainan Province and temperature spans −0.98 to 0.71, with a mean of −0.17. The area exhibiting a significant correlation between the carbon balance and temperature, as determined by the *t* test, constitutes 26.79% of Hainan’s total land area. Notably, 23.24% of this area shows a negative correlation, which is predominantly located in the north-central region of Hainan, encompassing areas such as Baisha Li Autonomous County, Danzhou City, and Chengmai County. The relationship between precipitation and the carbon balance in Hainan’s terrestrial ecosystems, as measured by the partial correlation coefficient, spans −0.99 to 0.80, with an average value of −0.18. A *t* test indicates that 28.27% of the region exhibits a significant correlation between the carbon balance and precipitation, with 25.07% displaying a negative correlation. These negatively correlated areas are situated primarily in southeastern Hainan, including Qiongzhong Li and Miao Autonomous County, Wuzhishan City, Qionghai City, Wanning City, and Sanya City. The influence of climate factors on the evolution of the carbon balance varies significantly spatially. In terms of average correlation coefficients and significant areas, precipitation has a slightly greater impact than temperature does, suggesting that it is the dominant meteorological factor influencing changes in the carbon balance in terrestrial ecosystems in Hainan Province.

Given the difficulty in quantifying the impact of human activities on the carbon balance in terrestrial ecosystems at the grid scale, in this study, residual trend analysis is used to obtain the carbon balance under climate conditions (CB_CC_) and under human activity (CB_HA_), classifying the trend of the carbon balance under human activity into five categories (Figures 6E and 6F). The areas with extremely significant and significant increases constitute 23.83% of the total area of Hainan Island; these areas are located primarily in the northwest coastal region, including the western part of Dongfang City, the northern part of Changjiang Li Autonomous County, the northwest part of Danzhou City, and Lingao County, as well as in the eastern part of Hainan Island, such as the eastern part of Chengmai County, the northern part of Ding’an County, the eastern part of Wenchang City, and the eastern part of Qionghai City. The areas with extremely significant and significant decreases comprise 16.80% of the total area of Hainan Island, which primarily surrounds the central mountainous region and the northeastern coastal area, including Haikou City. The areas with no significant increase or decrease constitute 59.37% of the total area of Hainan Island; these areas are located primarily in the central region, including Baisha Li Autonomous County, Qiongzhong Li and Miao Autonomous County, and Wuzhishan City.

#### Relative contributions of climate change and human activities to carbon balance changes

**Figure 7:**
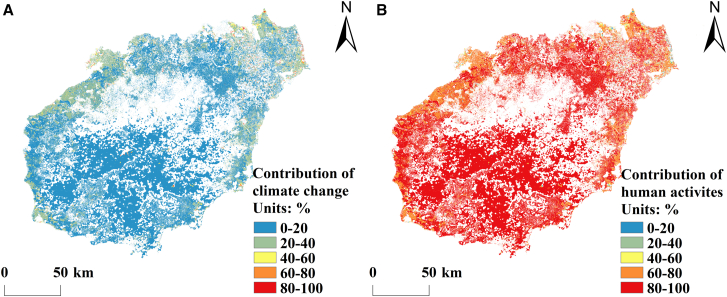
Spatial distribution of the contributions of human activities and climate change to improving the carbon balance on Hainan Island (A) Spatial distribution of the contribution of climate change to improving the carbon balance on Hainan Island. (B) Spatial distribution of the contribution of human activities to improving the carbon balance on Hainan Island.

**Figure 8:**
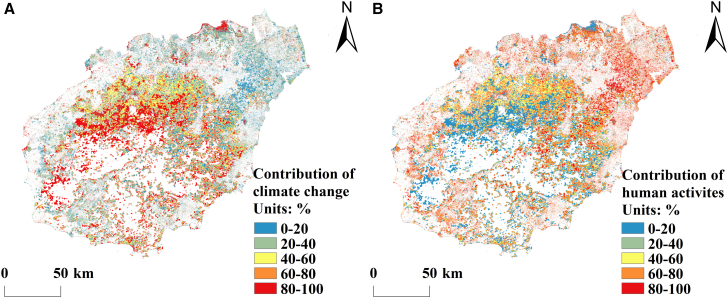
Spatial distribution of the contributions of human activities and climate change to inhibiting carbon balance on Hainan Island (A) Spatial distribution of the contribution of climate change to inhibiting carbon balance on Hainan Island. (B) Spatial distribution of the contribution of human activities to inhibiting carbon balance on Hainan Island.

The carbon balance changes in terrestrial ecosystems in Hainan Province are shaped by both climate change and human activities. The areas where the carbon balance has improved constitute 54.60% of Hainan Island, whereas 45.40% of the area has experienced degradation. In this study, the relative contributions of climate change and human activities to carbon balance shifts from 2000 to 2022 are estimated by analyzing actual values, predicted values, and residual trends. The contributions are categorized into five levels (Figures 7 and 8). In regions with improved carbon balance, climate change accounts for 10.55% of the impact, whereas human activities contribute 89.45%. Notably, only 0.83% of the area exhibits climate change contribution rates between 80% and 100%, whereas 78.16% is within this range for human activities. These high-impact human activity zones are concentrated mainly in southwest and northeast Hainan, including Changjiang Li Autonomous County, Dongfang City, and Ledong Li Autonomous County (Figure 7). In areas where the carbon balance has deteriorated, climate change and human activities contribute 50.46% and 49.54%, respectively. The proportion of areas where climate change accounts for 80%–100% of the impact is 26.94%, and these areas are distributed mostly in central-northern Hainan Province, such as in northern Baisha Li Autonomous County, southern Danzhou City, and northern Qiongzhong Li and Miao Autonomous County. Moreover, 17.69% of the land area is dominated by contributions from human activity within the same range, mainly in northeastern and coastal Hainan, including Haikou City, Wenchang City, Ding’an County, and Qionghai City (Figure 8). Based on the overall trends in carbon balance from 2000 to 2022, the relative contributions of climate change and human activities are calculated as 28.67% and 71.33%, respectively. These findings highlight the dominant role of human activities in shaping the carbon balance dynamics in terrestrial ecosystems in Hainan.

#### Analysis of the influence of different factors driving the carbon balance

In this study, natural and human factors are integrated, and 12 indicators across six categories are selected: meteorology, topography, land use, vegetation, soil, and socioeconomic factors. GeoDetector is employed to analyze the influence of these factors on the carbon balance of the terrestrial ecosystems of Hainan Province (Table 6). Among these factors, land use (q = 0.595) and the NDVI (q = 0.577) have *q* values exceeding 0.5, making them the most significant factors affecting carbon balance changes in Hainan Province. By constructing the transfer matrix of land use from 2000 to 2022 (Table 7), it can be found that the total area of land use change in Hainan Province during the 23-year period is 6424.82 km^2^, with the most obvious change in forest, followed by cropland. From 2000 to 2022, the total area of forest transformed to other land use types is 3392.16 km^2^, and the total area transformed from other types to forest is 2475.53 km^2^, which is the land use type with the most significant change, while the total area of cropland transformed to other land use types is 2755.87 km^2^, and the total area transformed into cropland is 3489.96 km^2^, which is the land use type whose change is in the second place. This is consistent with the current land use situation in Hainan Province, where vegetation resources are abundant, rubber forests are widely spread, and most of the land use types are forests, croplands, and orchards, thus the changes in NDVI and land use types become the dominant factors influencing the changes in carbon balance.

**Table 6:** Carbon balance factors and their q values

Type	Variable name	Variable meaning	Multiyear average *q* value	Ranking
Meteorology	X1	Temperature	0.088	11
	X2	Precipitation	0.420	8
Topography	X3	Elevation	0.195	10
	X4	Slope	0.208	9
	X5	Aspect	0.018	12
Land Use	X6	Land use	0.595	1
Vegetation	X7	Vegetation type	0.488	4
	X8	NDVI	0.577	2
Soil	X9	Soil erosion	0.429	7
	X10	Soil type	0.483	5
Socioeconomic Factor	X11	Population	0.461	6
	X12	GDP	0.492	3

**Table 7:** Transfer matrix of land use

2000/km^2^	2022/km^2^							
	Cropland	Forest	Shrubland	Grassland	Water	Barren	Imprevious	Total
Cropland	7075.54	2434.87	0.08	4.03	56.04	0.43	260.43	9831.41
Forest	3312.95	19636.95	0.44	0.80	10.70	0.05	67.22	23029.12
Shrubland	0.15	8.01	1.15	0.00	0.00	0.00	0.00	9.31
Grassland	55.44	4.58	0.02	3.82	8.35	0.42	15.43	88.06
Water	93.25	26.13	0.02	0.46	393.73	0.11	17.06	530.77
Barren	21.86	1.69	0.00	1.18	1.10	1.77	2.98	30.58
Imprevious	6.31	0.25	0.00	0.06	11.89	0.02	343.51	362.04
Total	10565.50	22112.47	1.70	10.36	481.82	2.80	706.64	33881.29

**Figure 9:**
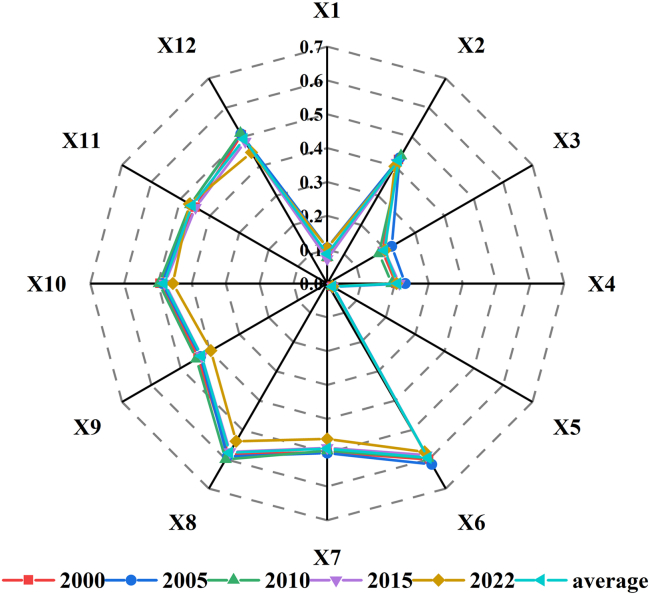
Detection of carbon balance factors by annual q values. X1–X12 represent temperature, precipitation, elevation, slope, aspect, land use, vegetation type, NDVI, soil erosion, soil type, population, and GDP, respectively

The average *q* values of GDP, vegetation types, soil types, population, soil erosion, and precipitation range from 0.4 to 0.5, indicating that these factors significantly influence the carbon balance changes in Hainan Province. These factors cover both natural and socioeconomic aspects and have relatively high impacts on the carbon balance. The soil type and soil erosion reflect the impacts of soil differences on the carbon storage capacity, whereas population and GDP represent the concentrated impacts of socioeconomic activities on the carbon balance. The *q* values for temperature, elevation, slope, and aspect are all less than 0.25, indicating a generally low influence on the carbon balance in Hainan Province. This is primarily due to the hilly and low mountainous terrain in Hainan Province, where most mountains range from 500 to 800 m in elevation. The annual average temperatures range from 22°C to 26 °C, with minimal variation between topographical and temperature factors, resulting in a lower influence on the carbon balance distribution. The diagram of the *q* values for each year reveals that the influence of each indicator on the carbon balance in Hainan Province remains relatively stable (Figure 9).

**Figure 10:**
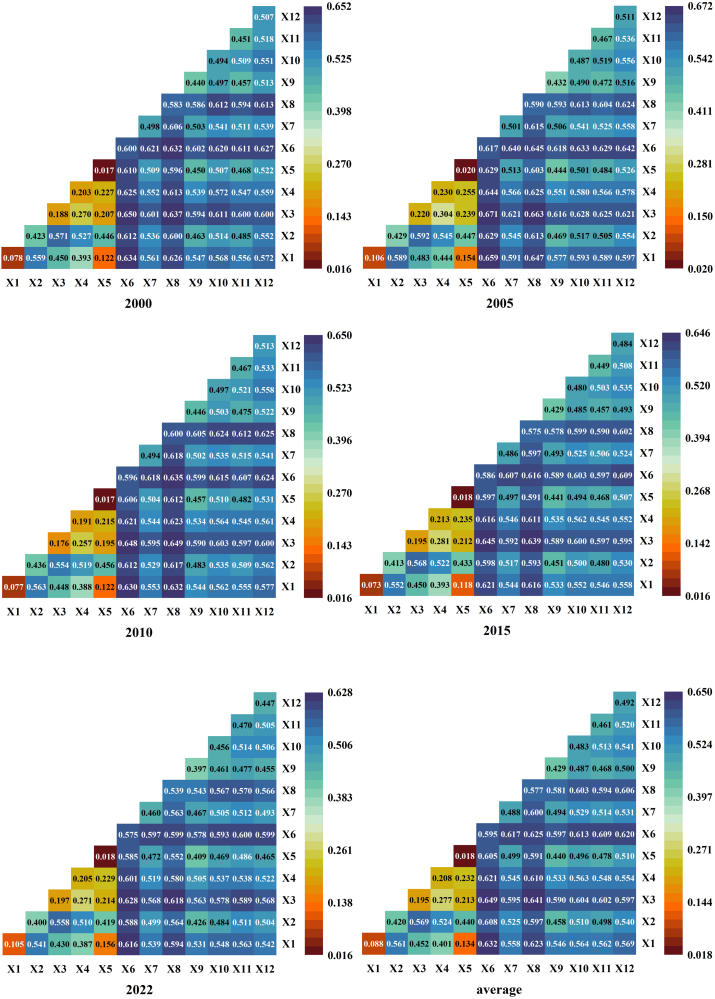
Interaction detection of carbon balance factors by annual q values X1–X12 represent temperature, precipitation, elevation, slope, aspect, land use, vegetation type, NDVI, soil erosion, soil type, population, and GDP, respectively.

The results from detecting the interactions among factors indicate that the explanatory power of factors for the carbon balance is significantly greater when two factors interact than when a single factor is considered, suggesting that the influences of different factors on the carbon balance in Hainan Province occur synergistically rather than independently (Figure 10). All interaction types are characterized by nonlinear and two-factor enhancements, with two-factor enhancement accounting for 87.8%, and nonlinear enhancement for 12.2%. On the basis of the multiyear average results, among the 12 driving factors, land use exhibits the greatest interaction with other natural and human factors, followed by the NDVI. In terms of specific indicators, the interaction between land use and elevation has the most significant influence on the carbon balance (q = 0.649), followed by the interaction between the NDVI and elevation (q = 0.641) and the interaction between land use and temperature (q = 0.632). This finding further indicates that the stronger the influence of a single factor on the carbon balance is, the greater its impact when it interacts with other factors. Conversely, a weaker single-factor influence can exert a stronger effect when interacting with other high-impact factors. Among the interactions with the least impact on the carbon balance are those between temperature and aspect (0.134), aspect and elevation (0.213), aspect and slope (0.232), and elevation and slope (0.277). This suggests that topographic factors share similar characteristics and that their synergistic impact on the carbon balance is relatively minor. When data across years are examined, the interaction between factors generally exerts a greater influence than do individual factors, with minimal variation between years, and the impact on the carbon balance remains relatively stable.

## Discussion

In this study, the InVEST model was used to assess the distribution of carbon storage in Hainan Province, and the carbon sequestration module in the model calculates carbon storage based on land use type and carbon density, which is of high accuracy and suitable for regions with complex land use types.^32^ The quantitative and scalable nature of the InVEST model has led to its wide application in carbon storage studies in Hainan Province and provided an important support for the simulation and causal analysis of carbon storage in Hainan Province.^18^^,^^32^ Between 2000 and 2022, the carbon balance in Hainan Province exhibits a fluctuating pattern of decline, followed by an increase and then another decline. Spatially, significant regional differences exist in the carbon balance of terrestrial ecosystems. High-value regions are concentrated predominantly in the central part of the island, whereas lower values are distributed mainly along the coastal perimeter, which aligns with the findings of Liu et al.^13^ The differences in the spatial distribution of the carbon balance in Hainan Province are related to its unique geographical location and ecological conditions. The central region of Hainan Province has high elevations, vast forest areas and includes various types of nature reserves and national forest parks. It is less affected by human activities, leading to a higher carbon storage capacity.^33^ In contrast, the coastal areas surrounding the island, characterized by lower elevations, are more susceptible to human-induced disturbances. These disturbances lead to changes in land use types and structures, thereby affecting the distribution of the carbon balance. The predominant land uses in these regions are agriculture and urban development, with a higher degree of urbanization than in the central region, which consequently results in reduced carbon storage. Human-induced land use changes are a key factor contributing to the divergent carbon balance distributions between the central region and the surrounding coastal areas of the island. Therefore, analyzing land use changes is essential for understanding the carbon balance.

Land use is a complex socioecological system whose evolution is influenced by human activities and whose comprehensive effects significantly impact human society.^13^ Differences in the land use type, area, and carbon density result in differential impacts of land use changes on carbon storage.^17^ The trend of the carbon balance in Hainan’s terrestrial ecosystems is influenced by shifts in land use patterns. Between 2005 and 2010, a significant upward trend is observed. This period coincides with the critical phase of China’s “11th Five-Year Plan”, during which Hainan prioritized increasing forest cover and enhancing its ecological environment. The key initiatives included large-scale afforestation projects, social forestry programs, and “greening around” measures, all of which significantly increased the province’s forested area. The forest cover increased from 55.6% to 60.2%.^17^ From 2010 to 2022, the carbon balance followed a fluctuating downward trajectory, closely linked to the rapid economic expansion after the establishment of the Special Economic Zone. Studies indicate a continuous annual increase in the construction land area across Hainan Province. Since the launch of the Hainan International Tourism Island policy in 2010, the area of construction land has increased by 122.46%.^13^^,^^34^ The dominant land use changes involve the transformation of forests into croplands, as well as the conversion of croplands and forests into construction land or water bodies.^34^ A reduction in the forest area can impact the local climate and atmospheric balance, which is a significant factor contributing to a decline in regional NEP. The expansion of construction land has altered the soil heterotrophic respiration rate in the area, reducing the regional NEP level and consequently causing corresponding changes in the carbon balance.^12^ Changes in land use types are the most direct manifestation of human activities, and their impact on the carbon balance reflects how human activities respond to changes in the carbon balance.

In addition to human-induced land use changes, climate change plays a crucial role in shaping the spatial distribution of the carbon balance in Hainan’s terrestrial ecosystems. While the contribution of climate change to the carbon balance is less pronounced than that of human activities are, its impact cannot be overlooked. The research results show that climate change has a negative effect on the carbon balance. This is because increased precipitation and warming have increased the productivity of the ecosystem, but this increase has been offset by the respiration of the ecosystem, resulting in a reduction in carbon sinks.^26^ Correlation analyses between the carbon balance and both temperature and precipitation reveal that precipitation has a broader impact, which aligns with the findings of Zhang et al. and Yue et al.^14^^,^^26^ Precipitation enhances vegetation growth, and China’s policy of converting farmland to forest and grassland has significantly increased the proportion of vegetation affected by precipitation in Hainan, thereby increasing forest carbon storage.^35^ However, since 2000, the rapid increase in carbon emissions has led to a continuous decline in the proportion of terrestrial carbon sinks offsetting carbon emissions. Compared with 30.97% from 1990 to 2000, the proportion decreased to 14.69% from 2010 to 2021, with the reduced part used to offset the increase in atmospheric CO_2_.^36^ Therefore, although the carbon sink of the ecosystem has increased, the growth rate of carbon emissions far exceeds that of the carbon sink, and ultimately, the change in precipitation has a negative effect on the terrestrial carbon balance. In addition, the distribution of the carbon balance is affected by temperature, although its area is smaller than that of precipitation. Temperature influences the carbon balance by altering the duration of vegetation growth and solar radiation absorption. Higher temperatures can extend the growing season of vegetation, thereby increasing productivity and photosynthetic efficiency. Increased solar radiation resulting from reduced cloud cover further enhances vegetation growth.^37^ While precipitation remains a key limiting factor for vegetation growth, a sustained rise in temperature could induce drought conditions that surpass the resilience threshold for vegetation, ultimately leading to vegetation degradation and consequently impacting the carbon storage capacity of the ecosystem.^37^^,^^38^ Related research indicates that from 2000 to 2015, the impacts of global warming were pronounced. Moderate increases in temperature positively influence net primary productivity (NPP) growth; however, sustained increases in temperature subsequently reduce vegetation productivity and increase soil respiration, ultimately diminishing the ecosystem’s carbon sequestration capacity.^11^ Overall, changes in the carbon balance are not attributable to a single factor but rather result from the cumulative effects of multiple factors, including climate change, land use alterations, and ecological engineering, which collectively determine the spatial distribution and dynamics of the carbon balance.

### Conclusion

Focusing on Hainan Province, in this study, the InVEST model and an enhanced NEP model are integrated to establish a framework for examining the spatial and temporal distributions of the carbon balance in terrestrial ecosystems. Correlation and residual analyses are utilized to quantify the respective impacts of climate change and human activities on variations in the carbon balance. Additionally, the GeoDetector model is employed to identify key factors driving changes in the carbon balance. (1) The expansion of carbon storage in Hainan significantly surpasses its reduction, with increases mainly concentrated in northern and northeastern cities. The distribution of NEP values reveals distinct spatial heterogeneity, indicating a strong overall carbon sink, whereas carbon source areas are located primarily in coastal urban centers. (2) The carbon balance in Hainan Province exhibits a fluctuating pattern, initially decreasing, then rising, and subsequently declining again. Forests have the highest average carbon balance values. Spatially, the carbon balance follows a central-high and peripheral-low distribution, forming a gradient increase from coastal to inland areas. (3) Climate change and human activities contribute 28.67% and 71.33%, respectively, to carbon balance shifts, highlighting the dominant role of human activities. Among all the influential factors, land use emerges as the most decisive. As a result, green planning and construction should be strengthened in areas with strong carbon sources, and greening areas should be increased to restore the function of carbon sinks. At the same time, it is necessary to minimize the interference of human activities with the carbon balance, reduce the degree of exploitation of land, and scientifically plan the production and development of land. By integrating multiple analytical approaches, this study provides insights into the carbon balance of terrestrial ecosystems and its drivers, offering guidance for regional carbon management and emission reduction strategies. Future studies should refine research objectives and further explore the mechanisms governing carbon balance transitions.

### Limitations of the study

This paper simulates the carbon balance distribution of Hainan Province by combining the InVEST model and the improved NEP model, and has achieved some results, but there are also limitations. First, the carbon density data used in this paper are based on previous studies and are applicable to most land use types in Hainan Province, but in fact, the land use types in Hainan Province are more complex, for example, for mangrove forests, salt marshes and other land use types, the lack of measured data on carbon density makes it impossible to reflect the actual situation in this range.^19^ Secondly, the transfer matrix of land use for 2000–2022 was used in analyzing the level of influence of different drivers on the carbon balance, but in reality, the land conversion in stages should also be taken into account, so as to provide a basis for analyzing the influence of land use on an ongoing basis. In future research, high-precision detection instruments and new technical means should be fully applied to obtain more field data for validation and use.

## Resource availability

### Lead contact

Further information and requests for resources and data should be directed to and will be fulfilled by the lead contact: Xiaobin Dong (xbdong@bnu.edu.cn).

### Materials availability

This study did not generate new unique reagents.

### Data and code availability

• This paper analyzes existing, publicly available data. These accession numbers for the datasets are listed in the key resources table.
• This paper does not report original code.
• Any additional information required to reanalyze the data reported in this paper is available from the lead contact upon request.

## Acknowledgments

This work was supported by the 10.13039/501100001809National Natural Science Foundation of China (no.42171275), the Specific Research Fund of The Innovation Platform for Academicians of Hainan Province (no.YSPTZX202308) and the Second Tibetan Plateau Scientific Expedition and Research Program (STEP) (no. 20190ZKK0608).

## Author contributions

Conceptualization: Q.Ye; data curation: Q.Ye, X.X., C.Z., Y.Y., Y.Q., R.X., and X.W.; formal analysis: Q.Ye, X.X., Y.Y., Y.Q., R.X., and X.W.; investigation: Q.Ye, X.X., and Q.Yu; methodology: Q.Ye and Q.Yu; software: Q.Ye and Q.Yu; writing-original draft: Q.Ye, X.X., Q.Yu, and C.Z.; validation: X.X., C.Z., Y.Y., Y.Q., X.W., and X.D.; visualization: Q.Yu, Y.Y., Y.Q. and R.X.; funding acquisition: X.D.; project administration: X.D.; resources: X.D.; supervision: X.D.; review and editing: Q.Ye and X.D. All authors commented on the final manuscript.

## Declaration of interests

The authors declare no competing interests.

## STAR★Methods

### Key resources table

**Table:** 

REAGENT or RESOURCE	SOURCE	IDENTIFIER
**Deposited data**		

Land Use/Land Cover	Landsat imagery	https://zenodo.org/records/12779975
Net primary production	NASA’s LP DAAC	https://lpdaac.usgs.gov/
Temperature	National Earth System Science Data Center	https://www.geodata.cn/main/face_science_detail?guid=164304785536614&typeName=face_science
Precipitation	National Earth System Science Data Center	https://www.geodata.cn/main/face_science_detail?guid=192891852410344&typeName=face_science
NDVI	Ecological Data Center of the Chinese Academy of Sciences	https://www.nesdc.org.cn/sdo/detail?id=60f68d757e28174f0e7d8d49
Vegetation type	Resource and Environmental Science Data Center of the Chinese Academy of Sciences	https://www.resdc.cn/data.aspx?DATAID=122
Soil type	Resource and Environmental Science Data Center of the Chinese Academy of Sciences	https://www.resdc.cn/data.aspx?DATAID=145
Soil erosion	Resource and Environmental Science Data Center of the Chinese Academy of Sciences	https://www.resdc.cn/data.aspx?DATAID=259
Soil organic carbon	Harmonized World Soil Database v2.0 (HWSD v2.0)	https://www.fao.org/soils-portal/data-hub/soil-maps-and-databases/harmonized-world-soil-database-v20/en/
Elevation	Geospatial Data Cloud	https://www.gscloud.cn/
Population	Oak Ridge National Laboratory under the U.S. Department of Energy	https://landscan.ornl.gov/
GDP	Resource and Environmental Science Data Center of the Chinese Academy of Sciences	https://www.resdc.cn/DOI/DOI.aspx?DOIID=33

**Software and algorithms**		

GIS software	ESRI	https://www.arcgis.com/index.html
The Integrated Valuation of Ecosystem Services and Tradeoffs (InVEST) model	Natural Capital Project	https://naturalcapitalproject.stanford.edu/software/invest
MATLAB	MathWorks	https://www.mathworks.com/products/matlab.html

### Method details

#### Research area

Hainan Island, which is situated at the southernmost tip of China (108°37′–111°03′E, 18°10′–20°10′N), covers a total area of 33,900 square kilometres and features a coastline extending 1,944.4 km, and it ranks as the country’s second-largest island (Figure 1). The region features a variety of landform types, characterized by higher elevations in the southwest and lower terrain in the northeast. Wuzhi Mountain (1,867 m) and Yingge Ridge (1,811 m) form the core of the uplifted region, with a topographic sequence transitioning from mountains and hills to plateaus and plains from the centre outwards.^39^ Hainan Island has a typical tropical monsoon maritime climate that is characterized by consistently high temperatures and substantial rainfall. The mean yearly temperature fluctuates between 23.8°C and 26.2°C, whereas the annual rainfall spans from 1,000 mm to 2,500 mm.^40^ Since Hainan was established as a province in 1988, and particularly after being designated an International Tourism Island in 2010, the region has experienced significant socioeconomic growth and rapid urbanization. These land use transformations have had a substantial influence on the island’s ecosystem structure and functionality. Presently, forests and cropland dominate land use on the island, collectively accounting for approximately 90% of its total area. The island is home to abundant flora and fauna, diverse ecosystems, and the best-preserved tropical natural forests in the country, providing a solid foundation for ecological development in Hainan Province and the construction of a national ecological civilization pilot zone.^41^^,^^42^

#### Data sources

The China Land Cover Annual Product (CLCD) for 2000–2022, derived from Landsat imagery, is available at https://zenodo.org/records/12779975. The CLCD includes nine land cover types: cropland, forest, shrubland, grassland, water bodies, ice/snow, barren land, impervious surfaces, and wetlands. In the context of the Hainan study area, land use classification on the basis of the CLCD dataset includes seven categories: cropland, forest, shrubland, grassland, water, barren land, and impervious surfaces.^43^

The net primary production (NPP) data for vegetation originate from the MODIS product MOD17A3HGF, which is available through NASA’s LP DAAC (https://lpdaac.usgs.gov/). The dataset employs the BIOME-BGC algorithm framework to compute daily Gross Primary Productivity (GPP) through a light-use efficiency model. Annual NPP values are subsequently generated by accumulating daily GPP, subtracting maintenance respiration losses across vegetation components, and applying a growth respiration optimization model. Algorithmic specifications are detailed in the product user manual.^44^ To process the raw data for analysis, MODIS Reprojection Tools (MRT) and geographic information system (GIS) software were used. The preprocessing steps involved mosaicking the images, converting the data format, reprojecting them into the geographic coordinate system, and applying the nearest neighbor interpolation method to preserve the 500 m spatial resolution. Finally, the dataset was clipped to match the boundaries of the study area. The application of the MODIS NPP product has proven reliable and is widely adopted across China.^45^^,^^46^^,^^47^

The average temperature and precipitation data for 2000–2022 were obtained from the National Earth System Science Data Center (https://www.geodata.cn), with a spatial resolution of 1 km. In this study, these datasets were used mainly to estimate soil heterotrophic respiration (Rh) in Hainan Province. Specifically, among these datasets, data from 2000, 2005, 2010, 2015, and 2022 were selected for analysis of driving factors using GeoDetector.

The NDVI dataset spanning 2000–2022 was acquired from the Ecological Data Center of the Chinese Academy of Sciences (https://www.nesdc.org.cn/). Derived from Landsat remote sensing data through the Google Earth Engine, it offers a spatial resolution of 30 m. Vegetation type data were obtained from the Resource and Environmental Science Data Center of the Chinese Academy of Sciences (https://www.resdc.cn/), with a spatial resolution of 1 km. This dataset classifies vegetation into 11 types, including broadleaf forests, coniferous forests, and mixed coniferous–broadleaf forests.

The soil factor dataset includes data on the soil type and erosion degree sourced from the Resource and Environmental Science Data Center of the Chinese Academy of Sciences (https://www.resdc.cn/) and has a spatial resolution of 1 km. Soil organic carbon data for the depth interval of 0–20 cm were extracted from the Harmonized World Soil Database v2.0 (HWSD v2.0), which is provided by the Food and Agriculture Organization (FAO) (https://www.fao.org). For China, the data originated from a scale of 1:1,000,000 soil dataset compiled by the Institute of Soil Science, Chinese Academy of Sciences, which is based on the Second National Land Survey and has a resolution of 1 km. Soil type maps were used to derive soil organic carbon density distributions. Elevation information was obtained from the Geospatial Data Cloud (https://www.gscloud.cn/), and slope and aspect data were computed using GIS software from elevation models. Both topographic datasets feature a spatial resolution of 30 m.

The socioeconomic dataset’s population data were derived from the LandScan Global Population Data Set, provided by the Oak Ridge National Laboratory under the U.S. Department of Energy (https://landscan.ornl.gov/), with a spatial resolution of 1 km. The accuracy of the LandScan dataset has been rigorously assessed at both the national and county scales.^48^^,^^49^^,^^50^ The GDP data originated from the Resource and Environmental Science Data Center of the Chinese Academy of Sciences (https://www.resdc.cn/). This dataset was developed through spatial interpolation of county-level GDP statistics, integrating spatial interactions among land use categories linked to human activities, nighttime light intensity, and settlement density. The resulting dataset was structured in a 1 km × 1 km grid format. The data applied in this study, along with their details, are summarized in Table 1.

In addition to the aforementioned spatial data, the carbon density data presented in Table 2, which were used for estimating carbon storage, were derived primarily from previous studies.^13^^,^^51^ Specifically, the carbon density data from Liu et al. were sourced from China’s systematic carbon density dataset, which compiles measurements from earlier studies.^13^^,^^52^ According to Liu et al., for Hainan Island, data points were selected based on geographical coverage, and the values were averaged to account for spatial variability.^13^ Additionally, to address the lack of carbon density data for barren land in certain areas, data from Fang et al. were utilized, which provided carbon density estimates specifically for barren land types in Hainan.^51^ These data serve as critical inputs for the InVEST model, which is designed to quantify and support the management of ecosystem services.^53^^,^^54^^,^^55^ The model has been widely applied to assess carbon storage across various ecosystems. It calculates carbon storage by multiplying the land area by the carbon density values for each land-use type, accounting for spatial variability.^56^^,^^57^

#### Assessment of the carbon balance in terrestrial ecosystems

In this study, the carbon balance of terrestrial ecosystems in Hainan Province is quantified by evaluating carbon storage and the NEP. Carbon storage encompasses aboveground and belowground biomass carbon, soil organic carbon, and dead organic matter carbon.^27^^,^^33^^,^^58^ Changes in carbon storage are driven primarily by shifts in land use types, while the effects of climate change are not accounted for in this analysis.^59^^,^^60^^,^^61^ In the model calculations, the carbon density of different land use types is assumed to be constant. Given the variability in carbon density among land use types, conversions between these types directly influence changes in carbon storage. Climate variables are incorporated to determine the NEP in the model, with climate change significantly affecting the NEP. To ensure unit consistency in the carbon balance calculation, NEP (originally expressed in Tg C/year) was aggregated over a one-year period for the selected study years, thereby converting NEP to a cumulative mass (Tg C). This harmonization enables the summation of carbon storage (Tg C) and cumulative NEP (Tg C) in the carbon balance equation. The formula for calculating the carbon balance of terrestrial ecosystems is as follows:^11^

(Equation 1) CB=CS+NEP×Δt

In the formula, *CB* denotes the carbon balance within the terrestrial ecosystems of the study area; *CS* indicates the total carbon storage across the study area; and *NEP* signifies net ecosystem productivity; Δt denotes the time interval (1 year) over which NEP is aggregated, resulting in a cumulative mass (Tg C) for the specified years.

#### Estimation of carbon storage using the InVEST model

In this study, the carbon storage module of the InVEST model is used to estimate total carbon storage on Hainan Island. This module classifies carbon storage into four key carbon pools: aboveground biomass carbon (i.e., carbon contained in all living plant tissues above the soil), belowground biomass carbon (i.e., carbon stored in root systems), soil organic carbon, and dead organic matter carbon (i.e., carbon held in plant litter and decomposed wood). By integrating the average carbon density of different land use categories with their corresponding areas, the regional carbon storage can be calculated. The foundational data principle is outlined as follows:^62^

(Equation 2) $$CS=\sum_{i=1}^{n}A_{i}\times \left(C_{ai}+C_{bi}+C_{si}+C_{di}\right)$$

In the formula, *CS* denotes the total carbon storage in the study area; *i* indicates the specific land use type; *n* represents the total number of distinct land use types; *A*_*i*_ signifies the area covered by the *i*-th land use type; *C*_*ai*_, *C*_*bi*_, *C*_*si*_, and *C*_*di*_ represent the aboveground carbon density (Mg/hm2), belowground carbon density, soil organic carbon density, and dead organic matter carbon density of the *i*-th land use type, respectively.

#### Simulation of NEP

Net ecosystem productivity (*NEP*) was first proposed by Woodwell et al. and is defined as the amount of net primary productivity (*NPP*) remaining after accounting for annual heterotrophic respiration (*Rh*).^63^ The details of the *NPP* data source and processing methods are provided in Section data sources. The formula for calculation is as follows:

(Equation 3) NEP=NPP−Rh

*Rs* represents annual soil respiration. Zhang et al. gathered 113 sets of measured *Rs* and *Rh* data from diverse regions across China.^64^ The dataset encompasses a broad spectrum of ecosystems, including forests, croplands, and grasslands, thereby offering robust regional representation. To investigate the relationship between *Rs* and *Rh*, multiple regression models were applied. The power function regression model was identified as the optimal fit, achieving an *R*^2^ of 0.758 and passing the significance test at the 0.01 level. The equation for estimating *Rh* is given below:

(Equation 4) $$Rh=0.6163Rs^{0.7918}$$

*Rs* is calculated using the model for simulating soil respiration developed by Chen et al.^65^ This model, which builds upon previous empirical models of soil respiration, incorporates key factors such as temperature, precipitation, and surface soil organic carbon storage. Consequently, it effectively elucidates the interannual and spatial variations in soil respiration. The *Rs* simulation model is formulated as follows:

(Equation 5) $$Rs=1.55e^{0.031T}\times \frac{P}{P+0.68}\times \frac{SOC}{SOC+2.23}$$

In the formula, *Rs* denotes the annual soil respiration rate (kg C m^–2^ yr^–1^), *T* denotes the annual mean temperature (°C), *P* denotes the annual precipitation (m), and *SOC* denotes the soil organic carbon density in the 0–20 cm layer (kg C m^–2^).

#### Method of trend analysis

To analyse the trend of carbon balance changes in Hainan Province’s terrestrial ecosystems, in this study, a simple linear regression model is employed,^11^^,^^66^^,^^67^ with calculations based on the following formula:

(Equation 6) $$\theta_{slope}=\frac{n\times \sum_{i=1}^{n}i\times CB_{i}-\sum_{i=1}^{n}i\sum_{i=1}^{n}CB_{i}}{n\times \sum_{i=1}^{n}i^{2}-{\left(\sum_{i=1}^{n}i\right)}^{2}}$$

In the formula, *θ*_*Slope*_ denotes the rate of change in the carbon balance. Specifically, a positive *θ*_*Slope*_ (*θ*_*Slope*_ > 0) indicates an upwards trend, whereas a negative *θ*_*Slope*_ (*θ*_*Slope*_ < 0) signifies a downwards trend. The variable *i* represents the year, where *n* equals 23, and *CB*_*i*_ denotes the carbon balance value for year *i*. By referencing the critical values from the *F*-distribution table, changes in the carbon balance trend can be categorized into five levels: extremely significant decreases (*θ*_*Slope*_ < 0, *P* < 0.01), significant decreases (*θ*_*Slope*_ < 0, 0.01 ≤ *P* < 0.05), no significant changes (*P* ≥ 0.05), significant increases (*θ*_*Slope*_ > 0, 0.01 ≤ *P* < 0.05), and extremely significant increases (*θ*_*Slope*_ > 0, *P* < 0.01).^66^ All statistical analyses were conducted using MATLAB 2018a based on per-grid values. Detailed statistical information is presented in the results section, specifically under the subsections titled “characteristics of changes in carbon storage and the NEP,” “temporal and spatial variations in carbon balance characteristics,” and “impact of climate change and human activities on the temporal and spatial evolution of the carbon balance,” with results visualized in Figures 3, 4, 5, and 6.

#### Correlation analysis

Correlation analysis is employed primarily to elucidate the magnitude and direction of relationships between variables.^14^^,^^35^ In practice, the relationship revealed by the simple correlation coefficient is usually affected by other variables, while the partial correlation coefficient can more accurately reflect the independent relationship between two variables after controlling for other related variables, and thus it is of more practical significance. In this study, to precisely determine the relationships between the carbon balance of terrestrial ecosystems and climatic factors, we calculated the partial correlation coefficient using the following formula:

(Equation 7) $$R_{xy}=\frac{\sum_{i=1}^{n}\left[\left(x_{i}-\bar{x}\right)\left(y_{i}-\bar{y}\right)\right]}{\sqrt{\sum_{i=1}^{n}{\left(x_{i}-\bar{x}\right)}^{2}\sum_{i=1}^{n}{\left(y_{i}-\bar{y}\right)}^{2}}}$$

In the formula, *R*_*xy*_ denotes the Pearson correlation coefficient between variables *x* and *y*; *x*_*i*_ represents the carbon balance value for year *i*; *y*_*i*_ represents the temperature or precipitation in year i;*¯x* and*¯y* denote the average annual carbon balance and corresponding average temperature and precipitation values for Hainan Province from 2000 to 2022, respectively; *i* is the index for that year; and *n* is the total sample size.

(Equation 8) $$R_{xy,z}=\frac{R_{xy}-R_{xz}R_{yz}}{\sqrt{\left(1-{R_{xz}}^{2}\right)}\sqrt{\left(1-{R_{yz}}^{2}\right)}}$$

In the formula, *R*_*xy,z*_ represents the partial correlation coefficient between *x* and *y* while controlling for a third variable *z*. In this context, the dependent variable is the carbon balance, whereas the independent variables are the average temperature and precipitation. The significance of the correlation coefficient is typically assessed using a *t* test, with the test statistic calculated according to the following expression:

(Equation 9) $$t=\frac{R_{xy,z}}{\sqrt{1-{R_{xy,z}}^{2}}}\sqrt{n-m-1}$$

In the formula, *R*_*xy,z*_ represents the partial correlation coefficient, where *n* denotes the number of years and *m* denotes the number of independent variables. The significance of the correlation coefficient is tested using significance levels of *α* = 0.05 and *α* = 0.01. Correlation strength is classified as follows: extremely significant positive correlation (*R*_*xy,z*_ > 0, *P* < 0.01), significant positive correlation (*R*_*xy,z*_ > 0, 0.01 ≤ *P* < 0.05), insignificant positive correlation (*R*_*xy,z*_ > 0, *P* ≥ 0.05), extremely significant negative correlation (*R*_*xy,z*_ < 0, *P* < 0.01), significant negative correlation (*R*_*xy,z*_ < 0, 0.01 ≤ *P* < 0.05), and insignificant negative correlation (*R*_*xy,z*_ < 0, *P* ≥ 0.05). All statistical analyses were conducted using MATLAB 2018a based on per-grid values. The corresponding statistical details are reported in the results section under “impact of climate change and human activities on the temporal and spatial evolution of the carbon balance,” with results visualized in Figure 5.

#### Residual trend analysis

In this study, residual analysis is applied to assess the distinct effects of climate change and human activities on the carbon balance.^68^ The process involves two key steps: (1) utilizing precipitation and temperature time series data as independent variables, a regression model is used to estimate the carbon balance value (*CB*_*CC*_), reflecting the influence of climate change; (2) the difference between the observed carbon balance value (*CB*_*obs*_) and the predicted *CB*_*CC*_ is calculated to obtain the residual (*CB*_*HA*_), which quantifies the impact of human activities on the carbon balance. The formula for the calculation is as follows:

(Equation 10) $$CB_{CC}=a\times T+b\times P+c$$

(Equation 11) $$CB_{HA}=CB_{obs}-CB_{CC}$$

In the formula, *CB*_*CC*_ represents the predicted carbon balance value derived from the regression model, whereas *CB*_*obs*_ denotes the actual observed carbon balance value. *CB*_*HA*_ signifies the residual. *T* and *P* represent the annual average temperature and precipitation, respectively, whereas *a*, *b*, and *c* are parameters within the regression model. By analysing the trend of residuals, in this study, the primary factors driving the carbon balance are categorized into six distinct categories. The detailed method of classification is presented in below Table.

**Table:** Identification criterion and calculation of the contribution of the drivers of carbon balance changes

Slope (*CB*_*obs*_)	Driving factors	Division criteria		Contribution rate/%	
		Slope (*CB*_*CC*_)	Slope (*CB*_*HA*_)	Climate change (CC)	Human activity (HA)
>0	CC & HA	>0	>0	$\frac{\text{slope}\left({\text{CB}}_{\text{CC}}\right)}{\text{slope}\left({\text{CB}}_{\text{obs}}\right)}$	$\frac{\text{slope}\left({\text{CB}}_{\text{HA}}\right)}{\text{slope}\left({\text{CB}}_{\text{obs}}\right)}$
	CC	>0	<0	100	0
	HA	<0	>0	0	100
<0	CC & HA	<0	<0	$\frac{\text{slope}\left({\text{CB}}_{\text{CC}}\right)}{\text{slope}\left({\text{CB}}_{\text{obs}}\right)}$	$\frac{\text{slope}\left({\text{CB}}_{\text{HA}}\right)}{\text{slope}\left({\text{CB}}_{\text{obs}}\right)}$
	CC	<0	>0	100	0
	HA	>0	<0	0	100

#### GeoDetector

GeoDetector is statistical analysis methods employed to identify spatial distribution differences and uncover potential driving factors.^35^^,^^69^^,^^70^ In this study, factor detectors and interaction detectors are used to examine the factors influencing the carbon balance in Hainan Province’s terrestrial ecosystems from 2000 to 2022. The formula for calculation is as follows:

(Equation 12) $$q=1-\frac{\sum_{h=1}^{L}N_{h}{\sigma_{h}}^{2}}{N\sigma^{2}}$$

In the formula, *N*_*h*_ and *N* represent the sample sizes for the h-th layer and the entire study area, respectively; *σ*_*h*_ and *σ* denote the variances in the carbon balance in the h-th layer and the overall study area, respectively; and *q* ranges from 0 to 1, where higher *q* values signify a greater influence of the factor on the carbon balance of Hainan Province’s terrestrial ecosystems, whereas lower *q* values indicate a lesser influence.
